# 65 Therapist Confidence Utilizing Virtual Range of Motion Methods

**DOI:** 10.1093/jbcr/irac012.068

**Published:** 2022-03-23

**Authors:** Miranda L Yelvington, Ingrid Parry, Michelle James, Kory Bettencourt, Sandra Taylor, David Greenhalgh

**Affiliations:** Arkansas Children’s Hospital, Little Rock, Arkansas; Shriners Hospitals for Children Northern California, Sacramento, California; Shriners Hospital for Children, Northern California, Sacramento, California; Shriners Hospital for Children, Northern California, Sacramento, California; University of California, Davis, Sacramento, California

## Abstract

**Introduction:**

Since the SARS-CoV-2 virus (COVID-19) was officially declared a pandemic, there has been a marked increase in virtual clinical care. Between 2019 and 2020, telehealth (TH) visits, including tele-rehabilitation (TR), increased from 11% to 46%. While many therapy interventions can be performed with verbal guidance or demonstration, objective tool-based outcomes such as goniometrics , a valuable tool to determine burn survivor progress, have proved more challenging. The purpose of this study was to evaluate the level of confidence of therapists using three different remote methods of measuring finger range of motion (ROM).

**Methods:**

Therapists evaluated finger ROM position of a mannequin model via a simulated TH visit using three different methods: Goniometry (GON), Visual Estimation (VE), and Electronic Protractor (EP). Pre and post-questionnaires were used to assess the participant’s experiences and comfort with each method of measurement. Descriptive statistics are used to report clinician opinions. A linear mixed effect model was used to determine the interaction of bias as a function of clinician characteristics (i.e., experience, familiarity, etc.).

**Results:**

A total of 30 therapists and one hand surgeon participated. All reported some (30%) or a lot (70%) of familiarity with standard GON, and most reported some (30%)or a lot (40%) of familiarity with finger-specific goniometry. Post-testing, clinicians reported VE (80%) as the most difficult method and EP (73%) as the easiest. Only 7% reported feeling more confident with TR compared to in-person measurements, 27% felt equally confident, and 67% felt less confident. The average time to conduct the remote assessment measurement was 11:45 minutes using GON, 4:27 minutes using VE and 9:47 minutes using EP. There was not a significant relationship between performance bias and years of experience (p=0.587), familiarity with GON (p=0.406), familiarity with finger GON (p=0.709) or profession (p=0.281).

**Conclusions:**

Despite the transition to virtual care, the mandate for valid and accurate documentation of functional outcome measures, including ROM, remains. Our study showed that the tools used for TR may not be the same as for in-person and clinicians need to adapt their approaches and skillsets. In addition, training with these new tools is essential for clinician confidence. In addition, there was not a relationship between experience and performance, suggesting that TR joint measurement is accessible to clinicians of all experience levels with proper training.

